# Unambiguous Linkage Between the Vaccination Coverage and the Spread of COVID-19: Geostatistical Evidence from the Slovak LAU 1 Regions

**DOI:** 10.1007/s41651-023-00144-2

**Published:** 2023-05-24

**Authors:** Branislav Bleha, Pavol Ďurček

**Affiliations:** Department of Economic and Social Geography, Demography and Territorial Development, Faculty of Natural Sciences, Comenius University in Bratislava, Bratislava, Slovakia

**Keywords:** COVID-19, Spatial analysis, Spatial spread, Vaccination coverage, Slovakia

## Abstract

This case study refutes some controversial findings about a minor connection between the vaccination coverage and the spread of COVID-19. We try to eliminate some methodological shortcomings and risks, which are included in such previously published studies. Firstly, our selection comprises all regional units in one country. Secondly, the quality of data is basically identical in all examined regions within the country. Thirdly, all Slovak regions had an equal starting position. They were at the same stages of the COVID-19 wave, and the measures taken were analogous in all regions. Slovakia with a significantly different vaccination rates among regions is a very suitable study case. We used the empirical data at the level of its LAU 1 regions for the two latest COVID-19 waves at that time (Delta, Omicron). The methods of regression analysis and geostatistical methods were applied in the study. Indubitably, there is an obvious link between the vaccination coverage and the spread of COVID-19. We have shown that the incidence-trajectories among regions vary based on the vaccination rates. The positivity and incidence in the most vaccinated regional populations were significantly lower than in the least vaccinated regions in a whole analyzed period. Their values in the best vaccinated regions were lower by roughly 20–25 % during the delta and omicron wave-peaks. Using the spatial autocorrelation, we also managed to clearly identify a close relationship between vaccination on the one hand and standardized incidence and positivity on the other hand, although some regions deviated from this general finding.

## Introduction and Background

As expected, the number of studies addressing various aspects of the COVID-19 proliferation has been growing very fast (Ye et al. 2021). These studies may be divided into the two fundamental groups. The first group has a medical, microbiological, and virological character, with several works clearly confirming that vaccination reduces the risk of Delta variant infection (Singanayagam et al. 2022; Levine-Tiefenbrun et al. 2021). In many cases, these studies are based on the measurement of viral load in samples and, subsequently, on proposals for an appropriate methodology to detect the relationships between viral load and transmission (Hay et al. 2021). Eyre et al. (2022) using a retrospective observational cohort conclude that vaccination is associated with a smaller reduction in the transmission of the Delta variant than the Alpha variant, and the effects of vaccination decrease over time, but the impact on reduction has remained.

The second type of studies utilizes mathematical, geostatistical, demographic, and geographical procedures. Recently, a multitude of such studies have been produced dealing with various attributes of COVID-19. However, only a smaller part of them are directly dedicated to measuring the effects of vaccination and to revealing the relationships between vaccination and the virus spread. The studies using mathematical approaches form one group. For instance, Yang et al. (2022) developed a sophisticated mathematical model concluding that, together with vaccination, reducing the contact rate of persons and increasing the isolation rate of infected individuals would substantially decrease the number of infections and shorten the time of the COVID-19 spread. This study belongs among those emphasizing the impact of vaccination if it is supported by other measures, especially epidemiological ones. As there were applied basically the same epidemiological measures in the entire territory of Slovakia, it was possible to measure the net effect of vaccination. Some other studies also devote themselves to mathematical modelling the COVID-19 spread, but not all of them are directly focused on the influence of vaccination on it (Kifle & Obsu 2022). Or, they are specially dedicated to the impacts of lockdowns and intervention strategies (Bugalia et al. 2020) and were developed at the time when vaccines were not available yet.

There is also a small group of studies that use geospatial modeling and geostatistics, in some cases related to demographic and population characteristics. Maza and Hierro (2021) analyze the transmission factors shaping the spatial distribution of COVID-19 infections during the distinct phases of the pandemic’s first wave in Madrid. They use a spatial regression model reflecting neighborhood effects among the municipalities. They focus on some geographical and demographic factors such as population, mobility, and some others. A study by Pizzuti et al. (2020) was produced already before the administration of vaccines; it modeled the spread of the virus using network-based prediction. Temerev et al. (2022) is an example of another study that uses the vaccination coverage for geospatial modeling.

Some complicated models try to capture the complex reality and background of the virus proliferation. However, such models may not well estimate the (isolated) effect of vaccination on the virus spread over space and time. Nevertheless, studies of this kind do exist, but their results are disputable. Our study reacts precisely to these works. Subramanian and Kumar (2021) completely doubt the impact of vaccination on the virus spread. For that reason, their study met with negative responses (e.g., Coleman et al. 2021; Gianicolo et al. 2021). It really used only trivial statistical procedures, but surprisingly — with such a methodological apparatus — was published in a prestigious epidemiological journal. Most likely, this work would not stand up in geographic or demographic journals.

But our study goes beyond brief, albeit true, critiques. It tries to show one of the possible ways of how such a case study could be performed. Its major contribution resides in original demonstration that the black-and-white vision does not exist in times of the COVID-19 pandemic either. The elimination of some methodological shortcomings and risks, which — in our opinion — are contained in simplified works, could be another asset of the study. Firstly, our selection includes all regional units in Slovakia. Secondly, the quality of data is basically the same in all examined regions of the country. Thirdly, all regions had an identical starting position within a general national framework, i.e., they were at the same stages of the COVID-19 wave, and the adopted measures were very similar in all Slovak regions, while countries of the world have been substantially differentiated from this aspect.

We would like to underline that even using all regional units, comparing only two 7-day averages as in case of US counties in the study by Subramanian and Kumar from 2021 is simply not enough. It is necessary to analyze a longer period, ideally from the beginning of the wave to its end. Our study deliberately deals with the spread of COVID-19 exclusively in regional populations. It does not address hospitalizations, numbers of patients on pulmonary ventilation, lethality, or mortality. These all are related phenomena, but their research requires a number of further methodological approaches. And the relationship between the virus proliferation and vaccination is very difficult to be clearly proven empirically. Finally, there is a large group of studies focused on the social and economic background and consequences of COVID-19 (Bidisha et al. 2021; Bukari et al. 2022).

## Data and Methods

Slovakia has recently been a suitable “laboratory” for research on the spread of the virus, also thanks to the strong regional disparities in the vaccination coverage. Its percentage values in several (LAU 1) districts exceeded 70%, whereas in some others were below 40%. Moreover, an increase in the vaccination coverage was negligible despite the culminating Omicron wave (roughly mid-February 2022). The LAU 1 regions also seem rather appropriate for analyses. In most cases, they approximately or partially coincide with the functional regions, i.e., the daily urban systems with the most intensive mobility and interactions of persons inside the units. In total, Slovakia consists of 79 LAU 1 units. Their use is a proper compromise between the efficient number of units, population size, and data availability. LAU 1 (formerly NUTS 4) is hierarchically higher statistical units joining LAU 2 (communes) together. The numbers of communes, the Slovak LAU 1 districts consist of, vary from 12 to more than 100, plus special urban units in the cities of Bratislava and Košice. What is also important, the LAU 1 borders were closed during the strict lockdowns. This means, the intra-regional vaccination rates matter more than inter-regional mobility and dispersion of the virus from one region to another.

Slovakia was affected considerably in both waves. The overall number of deaths is reported to exceed 20 thousand (April 2023). The total number of laboratory tests performed currently approaches 7.5 million, of which 1.7 million were positive. Mainly, the mRNAs (those from Pfizer and Moderna) were applied, and 97% out of all doses were these two types. Thus, the type of vaccine probably does not play a role in interregional differences.

Then, all data come from the National Health Information Centre (NCZI). All primary data are available also on the website korona.gov.sk. Firstly, we used correlation and regression analysis procedures, in which the vaccination of population (those fully vaccinated) in percent is the independent variable. The 7-day incidence from PCR tests (per 100,000 inhabitants) and the positivity of tests (in per cent) were used as the dependent variables. Here, the 7-day rolling averages were applied. In principle, given the availability of primary data from the PCR testing, there is nothing better for making analyses. However, we added also the rate of standardized incidence, which considers different levels and fluctuations in the volume of PCR tests carried out in the individual regions. The calculation works on the principle of weighting the regional share of PCR tests, using the proportion of inhabitants of the respective region in the total. It means that in those regions where the extent of testing was above average, the number of identified positive cases was lower. On the contrary, in those regions where the extent of testing was below average, the number of positive cases practically increased. The corresponding mathematical expression is as follows:

1 $$st{I}_i=\frac{stT_i^{pos}}{P_i}.100\ 000$$

2 $${stT}_i^{pos}={T}_i^{pos}.\frac{P_i}{\sum_{i=1}^n{P}_i}/\frac{T_i^{all}}{\sum_{i=1}^n{T}_i^{all}}$$

where

*stI*_*i*_ — standardized incidence in the region *i*

*stT*_*i*_^*pos*^— standardized number of positive PCR tests in the region *i*

*T*_*i*_^*pos*^— number of positive PCR tests in the region *i*

*stT*_*i*_^*all*^— overall number of PCR tests carried out in the region *i*

*P*_*i*_ — number of inhabitants in the region *i*

The standardization was done separately for each respective date.

Secondly, we separately analyzed two time series corresponding to the third (Delta) and fourth (Omicron) waves. September 1, 2021 can be identified as the beginning of the third COVID-19 wave in Slovakia. Already, the end of August of that year showed signs of a modest growth in the number of patients, but the September values may be considered more significant. While the Delta wave reached the peak during December 1–16, 2021, its end came approximately in mid-January 2022. The Omicron wave began soon — the starting date in the analysis is January 16, 2022, its peak was roughly in mid-February. Instead of analyzing the set of 79 units like in the first approach, we classified the LAU 1 units into the three categories, namely tertiles, for a better interpretation. The classification was based on the share of vaccinated persons in the total population on the respective dates. It reduced possible distortions associated with the different pace of vaccination in the regions from September 2021 to January 2022. If the vaccination level matters, the most telling value may likely be found at the beginning of the wave. We also tried a classification based on the values from the peak of the wave, but the results were essentially unaffected. In terms of vaccination, the position of the units within one wave was relatively stable.

The districts were also categorized alternatively as follows: the least vaccinated districts (30–40% of vaccinated persons), those with 40–50% of such persons, those with 50–60%, and the most vaccinated districts (60–70% of vaccinated persons). For the Omicron wave, the intervals were increased by 5% (35–45%, 45–55%, 55–65%, 65% and more, respectively), and the districts were re-grouped. However, the problem with this classification resided in the fact that the numbers of districts in each category were too different. The number of the most vaccinated districts with at least 60% of vaccinated persons was simply too low compared to the number of districts with less than 40% of those vaccinated. Although the differences between the most and least vaccinated regional populations were sizeable, we did not include these results due to statistical significance.

The third method we used is the hot-spot analysis — a local version of spatial autocorrelation (Getis & Ord 1992). This procedure, when applied to incidence, positivity and standardized incidence, allows identifying the extreme positions related to the virus spread at various rates of significance — that is, those regions where the situation was the worst or the best. At the same time, we used the hot-spot analysis also for the identification of regions where the largest deviations from the values of the regression model were achieved. This too served to describe the spatial variability of the COVID-19 spread. Likewise, we used the hot-spot analysis since one of its basic properties is the ability to identify only those high or low values that may be found — besides the region concerned — also in neighboring regions. This is the essence of the spatial autocorrelation procedure (Bleha & Ďurček 2019). The analysis of residuals was carried out as well.

The results in the form of residuals (more in the “Results and Discussion” section) allowed the application of the last method, namely geographically weighted regression (GWR). The main reason for using the GWR model is the fact that the basic ordinary least squares (OLS) model is unable to capture the existence of spatial differences for two or more variables. That means that through the GWR method, we are able to measure these spatial differences, especially in the clusters of residuals. GWR is a local form of linear regression calculated for each unit and the nearest neighbors. Thus, the model can be written as:

3 $${y}_i={\beta}_0\left({u}_i;{v}_i\right)+\sum_j{\beta}_j\left({u}_i;{v}_i\right){Xx}_{ji}+{\varepsilon}_i$$

where

*y*_*i*_ is the dependent variable.

*X*_*ji*_ is the *jth* independent variable.

*βj (ui,v*_*i*_ *)* is the *jth* coefficient at location *(u*_*i*_*,v*_*i*_*).*

*ε*_*i*_ is the random error term.

Unlike OLS, the parameters are allowed to vary by location *(u*_*i*_*,v*_*i*_*).*

Defining the neighborhood represents the most challenging point in the whole GWR analysis. The two basic possibilities of defining are the so-called fixed kernel and the adaptive kernel (Sachdeva & Fotheringham 2020). After testing several variants of defining the neighborhood (more also in Brunsdon et al. 1996; Haining 2003; Smith et al. 2018; Horák 2019; Horák et al. 2020), we chose the fixed kernel — here, we included 20 nearest neighbors for each unit. We used software ArcGis, namely the tool “Geographically Weighted Regression,” to calculate GWR. The global value of *R*^2^ or *adjR*^2^ was calculated as the average of local results in the individual regional units. The AICc indicator was used to compare the models that have a different dependent variable. The model with a lower AICc value provided a better fit to the observed data.

## Results and Discussion

Table 1 demonstrates the basic results of the statistical analysis in the selected time sections. The trend of indicators is evident. With the rising Delta wave during October and November 2021, there was a growing indirect dependence between the share of vaccinated persons and positivity, and also between the share of vaccinated persons and standardized incidence.

**Table 1:** OLS regression analysis (percentage of fully vaccinated persons as the independent variable)

Dependent variables ↓	*n*	*x*	*s*	*r*	*F*	*r*^2^	_*adj*_*r*^2^	*b*_0_	*Low*_*b*0_	*Up*_*b*0_	*b*_1_	*P*_*b*1_	*Low*_*b*1_	*Up*_*b*1_
Incidence Sept. 1, 2021 (start of Delta wave)	79	1.99	1.55	0.17	0.13	0.03	0.02	0.41	− 11.47	17.27	0.04	0.13	− 1.64	2.47
Incidence Dec. 1, 2021 (peak of Delta wave)	79	140.48	82.48	0.18	0.11	0.03	0.02	97.13	42.71	151.56	1.01	0.11	− 0.24	2.26
Incidence Jan. 16, 2022 (start of Omicron wave)	79	42.17	25.94	0.13	0.26	0.02	0.00	41.39	38.20	44.58	0.04	0.26	− 0.03	0.10
Incidence Feb. 16, 2022 (peak of Omicron wave)	79	309.00	83.00	0.19	0.09	0.04	0.02	201.96	103.55	321.26	2.21	0.09	− 0.34	4.66
Positivity Sept. 1, 2021 (start of Delta wave)	79	3.18	2.66	− 0.06	0.61	0.00	0.00	4.09	0.53	7.66	− 0.02	0.61	− 0.10	0.06
Positivity Dec. 1, (peak of Delta wave)	79	36.10	6.34	− 0.50	0.00	0.25	0.24	56.34	48.98	63.70	− 0.43	0.00	− 0.60	− 0.26
Positivity Jan. 16, 2022 (start of Omicron wave)	79	25.25	7.60	− 0.26	0.02	0.07	0.05	49.24	43.60	54.88	− 0.25	0.02	− 0.46	− 0.04
Positivity Feb. 16, 2022 (peak of Omicron wave)	79	59.40	6.20	− 0.47	0.00	0.23	0.22	79.45	71.06	85.63	− 0.41	0.00	− 0.58	− 0.25
Standardized incidence Sept. 1, 2021 (start of Delta wave)	79	2.13	1.63	− 0.11	0.32	0.01	0.00	3.22	7.27	37.87	− 0.03	0.32	− 0.08	0.02
Standardized incidence Dec. 1, 2021 (peak of Delta wave)	79	142.01	46.12	− 0.49	0.00	0.24	0.23	211.52	184.38	238.67	− 1.54	0.00	− 2.16	− 0.92
Standardized incidence Jan. 16, 2022 (start of Omicron wave)	79	40.51	11.62	− 0.22	0.05	0.05	0.04	48.70	42.78	54.62	− 0.14	0.05	− 0.28	0.00
Standardized incidence Feb. 16, 2022 (peak of Omicron wave)	79	30.70	31.00	− 0.44	0.00	0.20	0.19	396.98	356.63	437.34	− 1.86	0.00	− 2.71	− 1.01

The coefficient of determination indicates that more and more variability of indicators over time may be explained through the vaccination rate when the wave was rising. This is largely true for more suitable indicators such as positivity and standardized incidence. To increase the objectivity of the analysis, we also present the adjusted coefficient of determination, which reflects the number of statistical units. However, its values do not differ much from an unadjusted version. Intercept *b*_0_ shows the values of indicators in a hypothetical case, if each Slovak region achieved a zero level of vaccination (no vaccines were available). The slope *b*_1_ indicator demonstrates a theoretical change in the values, if the vaccination rate increased by 1 percentage point against the current value. Thus, a higher vaccination coverage would significantly reduce both positivity and incidence values in the model. As regards the Delta wave, the model predicts that in case of 65% vaccination, the current values of positivity would be roughly 20% instead of more than 30%. Moreover, 65% is still a value considerably below the values in the most vaccinated countries. With respect to the Delta wave, if the half of less vaccinated regional populations had been virtually “removed” from the total Slovak population, the country would have been in the better half in the European ranking of the virus spread instead of being in the worst quartile. Within the Omicron wave, the values of correlation and determination are only slightly lower. Despite the quicker and easier spread of the Omicron variant in populations, vaccination seems to be an effective “preventer.”

*n* count, *x* mean, *s* standard deviation, *r* Pearson’s correlation coefficient, *r*^*2*^ coefficient of determination (*R* square), *adjr*^*2*^ adjusted coefficient of determination, *F* significance level of correlation coefficient, *b*_*0*_ intercept, *P*_*b0*_ significance level of coefficient *b*_0_, *Low*_*b0*_ lower bound of the 95% confidence interval of coefficient *b*_0_, *U*_*pb0*_ upper bound of the 95% confidence interval of coefficient *b*_0_, *b*_*1*_ slope, *P*_*b1*_ significance level of coefficient *b*_0_, *Low*_*b1*_ lower bound of the 95% confidence interval of coefficient b1, *Up*_*b1*_ upper bound of the 95% confidence interval of coefficient *b*_1_

**Fig. 1:**
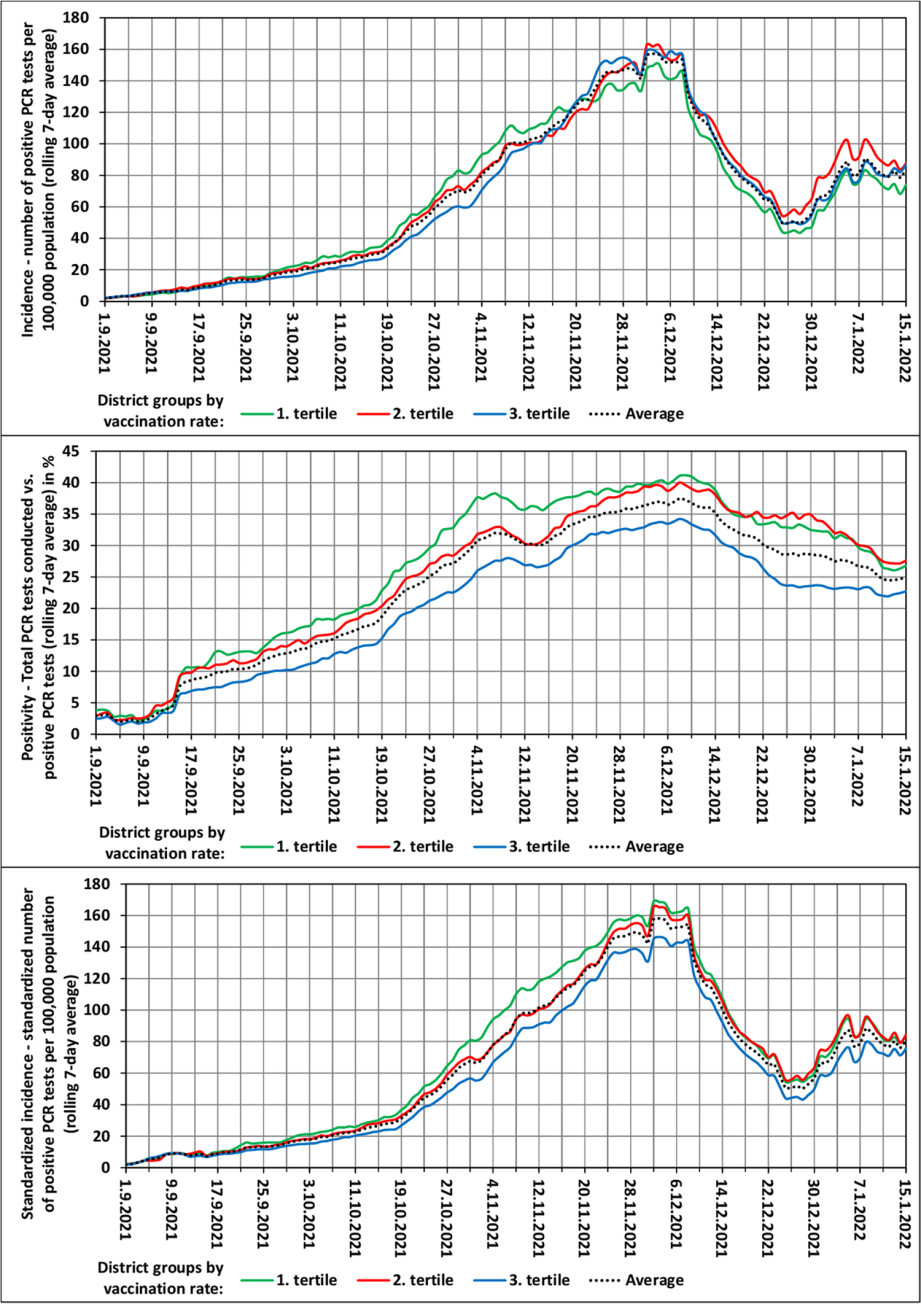
Delta wave characteristics in the groups of districts by rate of vaccination. 1st tertile is least vaccinated group of districts; 3rd tertile is most vaccinated group of districts

Figure 1 depicts the development of the three dependent variables over time, with the LAU 1 regions arranged into three tertiles according to the rate of vaccination in the Delta wave. All three indicators show a gradual “scissors opening” between the groups of more and less vaccinated regional populations. The greatest divergence is manifested in positive PCR tests. The only exception is formed by non-standardized incidence, the latest values of which do not differ much. This indicator is gradually losing its explanatory power and ability to demonstrate the variability. Concerning rate of positivity and standardized incidence, there is an evident gap between the group of the most vaccinated regional populations and the other groups. The difference is most pronounced at the peak of the wave.

**Fig. 2:**
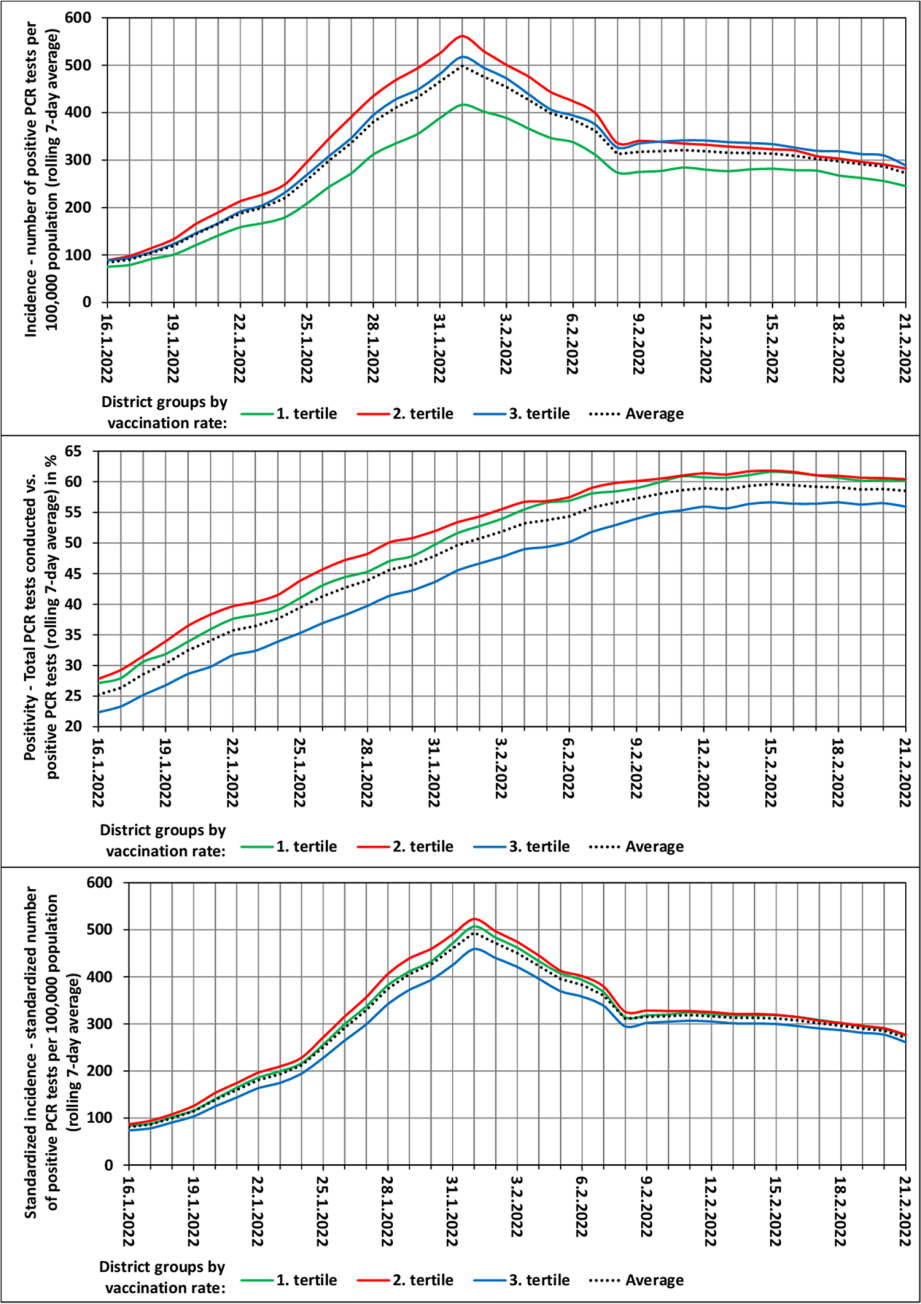
Omicron wave characteristics in the groups of districts by rate of vaccination. 1st tertile is least vaccinated group of districts; 3rd tertile is most vaccinated group of districts

Figure 2 depicts the development of the three dependent variables over time, with the LAU 1 regions arranged into three tertiles according to the rate of vaccination in the Omicron wave. A comparison of the curves in Figs. 1 and 2 suggests that vaccination in the Omicron wave had slightly a lesser effect on inter-regional differences. But even here, the impact is not negligible, particularly as regards a gap between one-third of the most vaccinated units and the other units.

**Fig. 3:**
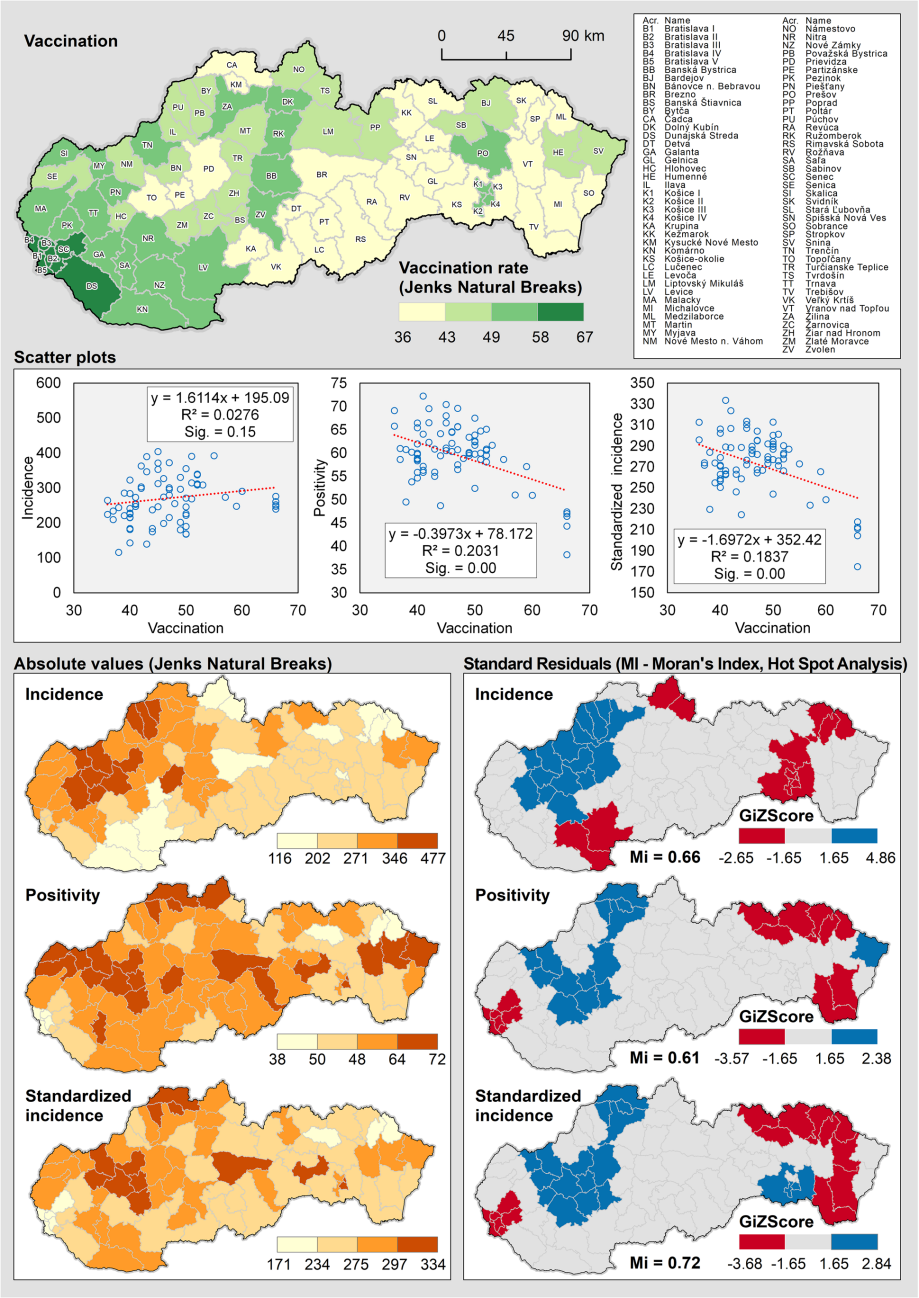
The spatial distribution of observed indicators and the spatial autocorrelation of residuals from the OLS model in the set of LAU 1 regions (Omicron wave)

**Fig. 4:**
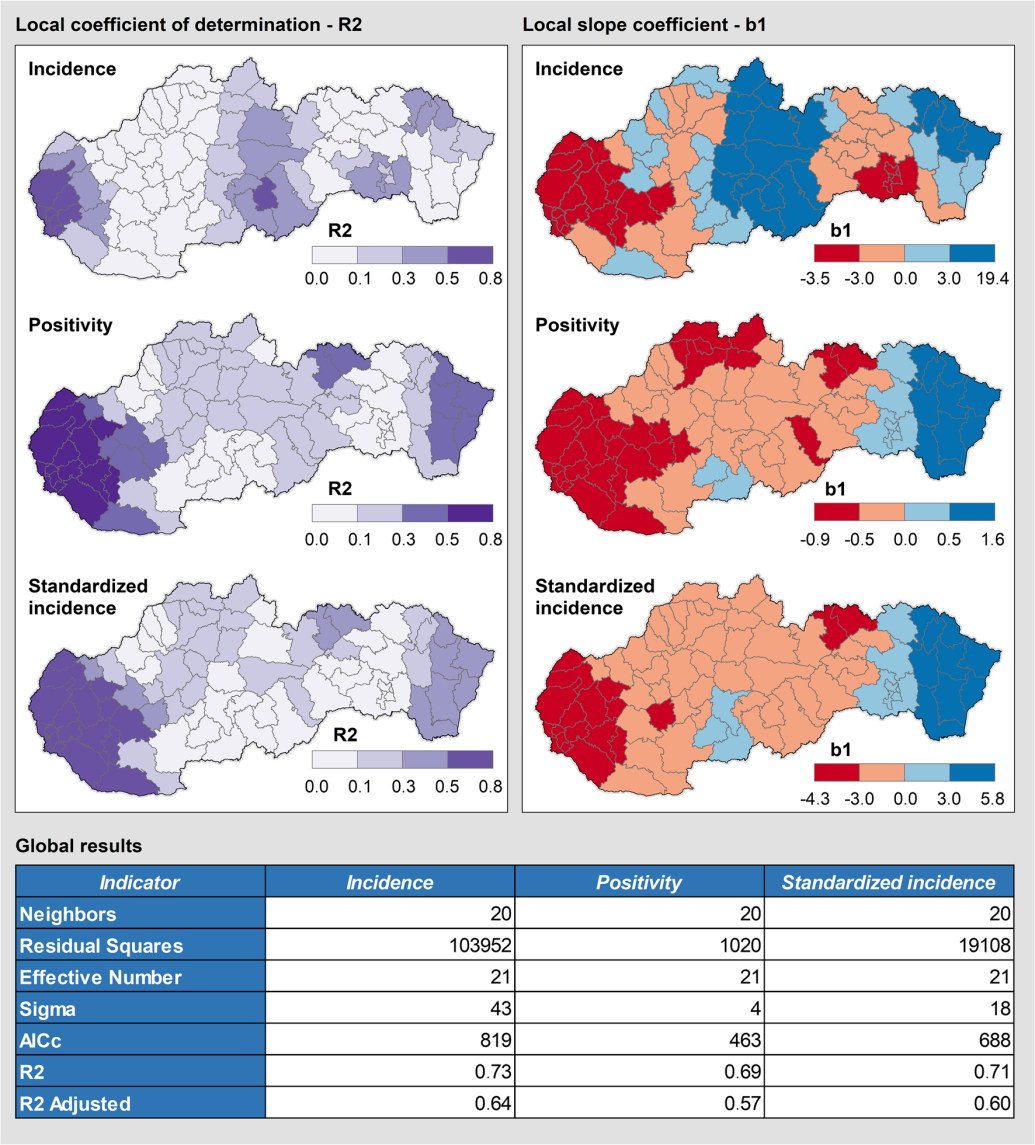
Main results of the GWR method in the set of LAU 1 regions (Omicron wave)

Opponents of the effect of vaccination on the virus spread and on inter-regional differences in positivity and incidence might not be convinced by the values in Table 1 as well as Fig. 1 and 2. However, especially in the Delta wave, the values of statistical indicators are relatively high, surely not negligible. Nevertheless, there is certainly not a high correlation. Therefore, we went further and applied other two procedures (Figs. 3 and 4), which may reveal regional clusters where vaccination works more and less.

Figure 3 is a series of maps where the hot-spot spatial autocorrelation method was applied to a set of regions in Slovakia as of February 14, 2022 (i.e., at the crude peak of the Omicron wave). An interesting finding is documented by three maps on the right showing the residual values of the regression model. The maps offer an interpretation — the identification of districts that match our model within the 90% confidence interval (Std. Dev. V-1.65 to 1.65). They form a major part out of the 79 country’s districts, especially in the second and third maps on the right. In this majority group, we managed to clearly identify a close relationship between vaccination on the one hand and standardized incidence and positivity on the other hand.

There are several possible explanations of why some regions face a better/worse situation than the model-predicted values. Among others, the impact of the previous waves on regional populations could play an important role. There is still no robust dataset on the presence of antibodies from laboratories that would meet the criteria for a significant geostatistical analysis. Some factors may be classified as “behavioral” ones. From the sociological, economic, and demographic viewpoints, though being a small population unit, Slovakia is a very heterogeneous country with distinct regional disparities. An elementary, very crude, division comprises the two macro-regions, namely the “poor South-East” and the “rich North-West” (Korec 2014). This basically reflects the vaccination coverage, but it could also encompass aspects such as reluctance towards the measures and COVID-19 rules. The political parties with a strong anti-vax and anti-rules agenda have a stronger support in poorer regions with lower education levels as well as higher unemployment rates (Plešivčák et al. 2018). This could be manifested in a more difficult tracking of contacts and some other aspects resulting in an easier proliferation of the virus in respective regions. Below, we also discuss the possible impact of different declines in regional mobility.

The global values of *R*^2^ oscillate around 0.7 (Fig. 4). The adjusted values are slightly lower, but still significantly higher than those for normal regression (OLS) given in Table 1. The model best fits for the indicator of positivity, as evidenced by the lowest AICc value. The better GWR score compared to OLS unambiguously confirms that the relationship between vaccination and rates of the COVID-19 spread is generally strong, but varies considerably among the regions. A strong negative correlation (*R*^2^ > 0.5; *b*_1_ < 0) may be found in the western part of the country. The central part of Slovakia normally has a weak negative correlation (*R*^2^ < 0.3; *b*_1_ < 0). An interpretation of the figures could be that vaccination here helps prevent the spread of the virus less — and it seems that vaccination has no effect on incidence and positivity in the easternmost part of the country (*R*^2^ > 0.3; *b*_1_ > 0). This can be explained also by the fact that there was only a small decline in mobility in this area compared to the beginning of September 2021. This hypothesis is also supported by Community Mobility Reports data from Google. Indeed, the decline in mobility in eastern Slovakia was on average lower than in western Slovakia. On the other hand, the regional data on mobility comprise also several discrepancies, and its explanatory power is thus limited.

There is a great difference between the big cities and rural or underdeveloped areas with generally lower mobility, primarily the intensity of commuting. This means that high mobility is likely to negate the benefits of vaccination in slowing down the spread of the virus. However, other factors too need to be taken into account, particularly those that may cause a weakening of the link between vaccination and the COVID-19 proliferation in regional units. Among others, let us mention socio-demographic characteristics — for instance, the different shares of segregated Roma communities occurring mainly in the south of central Slovakia and in its east (Šprocha & Bleha 2018). In these areas, the low level of vaccination is associated with low labor mobility; on the other hand, the virus is easier to spread here due to high population density.

There are several possible explanatory schemes and factors. We have included some of them into Table 2, where at least a moderate degree of dependence is expressed by the Pearson coefficient. In general, the higher vaccinated regions are those with better social-economic status (wages, educational structure), and this is why the positivity was lower in such regions, but the values of Pearson’s coefficient were moderate, and dependency was significant but not very strong. Furthermore, some dependencies are stable over time, but some are fluctuating.

**Table 2:**
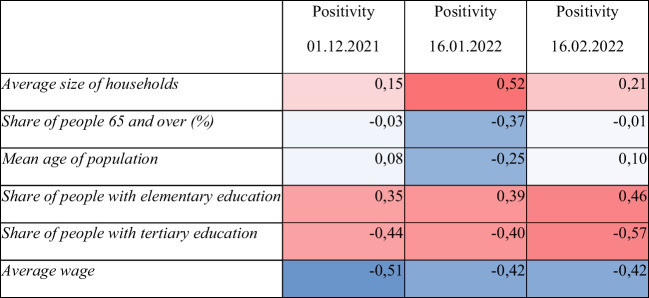
Pearson’s coefficient of correlation in set of LAU 1 units (positivity in % versus selected indicators)

The red cells represent a positive correlation. The blue cells represent a negative correlation. Deeper colours are used for higher values of correlation

## Conclusions

The results of our statistical and geostatistical analysis for the LAU 1 regions in Slovakia quite clearly indicate that the vaccination rates matter. The statistical dependence of indicators in the set of 79 units (i.e., country’s administrative districts) was undeniably increasing, the scissors between the most and least vaccinated regions in the values of incidence and positivity were opening, and this trend was lasting for two and a half months in the Delta wave. In terms of the Omicron wave, the results are roughly the same, depending on a concrete indicator. This is true despite facts such as a lower decrease in mobility, less strict measures, and weaker adherence to them. Because of their massive numbers, the tracing of contacts of infected persons was also a problem. In spite of that, even in the case of the Omicron wave, a significant effect was demonstrated using more elementary methods. It turns out that simpler methods too can have a telling value, if properly used and if data are consistent as well as robust enough. However, it is beneficial to add geostatistical approaches helping reveal the causes of lower correlation achieved by conventional statistics. This is especially true if there are not enough suitable indicators for a factor analysis, as in the case of this study.

The key message of the results is that a black-and-white vision of the vaccination effect on the virus spread in the population barely exists. Spatial methods disclose the real strength of the link between the vaccination coverage and positivity or incidence. Casting doubt on the impact of vaccination stands on shaky grounds. On the other side, presenting the results of maps with an almost perfect correlation between incidence and vaccination, for example in a set of European countries, is inappropriate too. Certainly, this link existed for hospitalizations and deaths to a substantial extent during the Delta wave; however, the different timing of the wave in various countries makes such comparisons inadequate. In regional populations, further attention should be paid to socio-demographic and geographical predictors increasing/decreasing the vaccination effect. By this, we mean not only age, but also the level of education, mobility, willingness to respect anti-pandemic rules, and several other factors.
